# Ophthalmic Manifestations of Hematopoietic Malignancy

**DOI:** 10.1155/2016/6074968

**Published:** 2016-06-07

**Authors:** Natsuyo Yoshida-Hata, Naomichi Katai, Toshiyuki Oshitari

**Affiliations:** ^1^Department of Ophthalmology, National Center for Global Health and Medicine, 1-21-1 Toyama, Shinjuku-ku, Tokyo 162-8655, Japan; ^2^Department of Ophthalmology and Visual Science, Chiba University Graduate School of Medicine, Chiba, Japan

## Abstract

*Purpose*. To report the ocular findings in patients with hematopoietic malignancy with optic nerve involvement and abducens nerve palsy.* Methods*. The medical records of all cases of hematopoietic cancer with ophthalmic involvements seen in the Department of Ophthalmology of the National Center for Global Health and Medicine between 2009 and 2014 were reviewed.* Results*. Eight patients with hematopoietic cancer with optic nerve invasion or abducens nerve palsy were studied. The primary diseases were 3 cases of multiple myeloma, 1 case of acute lymphocytic leukemia, 1 case of follicular lymphoma, and 3 cases of AIDS-related lymphoma. Six cases had optic nerve invasion, 2 cases had abducens nerve palsy, and 1 case had optic nerve invasion of both eyes. The median visual acuity of eyes with optic nerve invasion was 0.885 logarithm of the minimum angle of resolution (logMAR) units. The final visual acuity of eyes with optic nerve invasion was 1.25 logMAR units, and that of those with sixth-nerve palsy was −0.1 logMAR units. Six cases died during the five-year follow-up period. An ophthalmic involvement in patients with hematopoietic cancer, especially AIDS-related lymphoma, was associated with poor prognosis.* Conclusion*. Because ophthalmic involvement in patients with hematopoietic malignancy has a poor prognosis, an early diagnosis of the cancers by the ophthalmologic findings by ophthalmologists could improve the prognosis.

## 1. Introduction

The opportunity of ophthalmologists to see patients with hematopoietic cancer accompanied by a metastasis to the central nervous system (CNS) has increased [1, 2]. A recent review of patients with multiple myeloma showed that the median survival time had increased from 3 to 6 years in the past two decades [3]. Orbital infiltrations in patients with hematopoietic cancer are still rare with about 44 cases reported in the literature [1, 4–16]. The findings in the past cases showed that the incidence of CNS involvement by multiple myeloma was not frequent [13]. The incidence of orbital involvement in patients with multiple myeloma appeared to be less than that of CNS involvement. The number of the reports regarding ophthalmic manifestation in hematological malignancy was limited.

Our review of the clinical records of all hematopoietic patients who were examined in the Department of Ophthalmology, National Center for Global Health and Medicine Hospital, included 8 patients. The purpose of this study was to determine the clinical features of the optic nerve and abducens nerve in these 8 patients with hematopoietic cancer.

## 2. Case Presentation

We reviewed the medical records of all hematopoietic patients with ophthalmic manifestations who were examined between 2009 and 2014. This was a case control retrospective study, and eight patients who were diagnosed with hematopoietic cancer and had orbital involvement (Table 1) were studied. The follow-up period ranged from 1 month to 5 years.

The research protocol was approved by the Institutional Review Board, and the procedures were carried out in accordance with the tenets of the Declaration of Helsinki. A written informed consent was obtained from all participants for the treatments and the use of the medical information contained in their medical records.

### 2.1. Representative Case: Case  1

A 52-year-old man with HIV and AIDS-related lymphoma (ARL) presented with a 1-week history of acute vision reduction in the left eye. At that time, he was undergoing extensive antiretroviral therapy and chemotherapy for an AIDS-related lymphoma (ARL) associated diffuse large B cell lymphoma. The lymphoma was believed to be progressive because he had received intrathecal cytarabine and six cycles of cyclophosphamide, doxorubicin, vincristine, and prednisone with no sign of regression.

**Figure 1:**
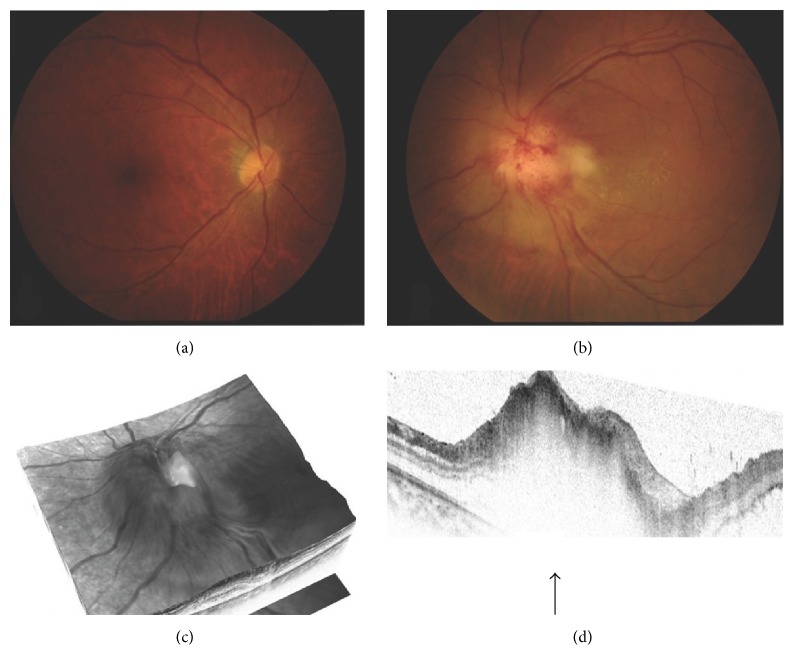
Fundus photographs and optical coherence tomographic (OCT) images of a patient with hematopoietic malignancy. ((a) and (b)) Color fundus photographs show severe left optic disc elevation with peripapillary hemorrhages and subretinal fluid. ((c) and (d)) OCT images of the left eye. The black arrow points to the optic disc.

His left eye had a relative afferent pupillary defect, and the decimal visual acuity was 0.05. The anterior chamber and lens were normal. The optic disc was markedly swollen with a yellowish-white thickening. There were also peripapillary hemorrhages, serous retinal detachment surrounding the optic disc, and engorged vessels in the left eye (Figures 1(a) and 1(b)).

**Figure 2:**
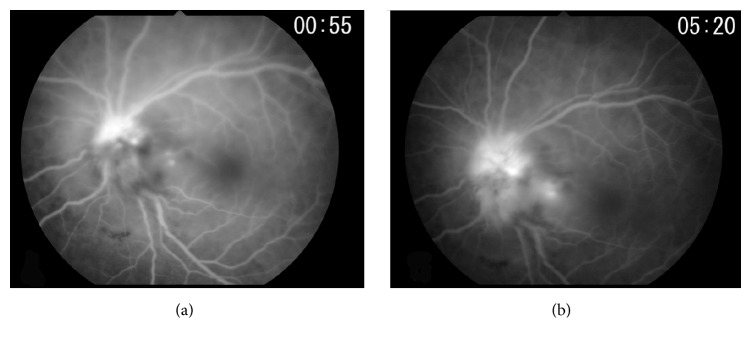
Fluorescein angiograms (FA) at the early phase (a) and late phase (b). FA images show subretinal fluid during the early phase (a) that is still present in the late stage (b).

**Figure 3:**
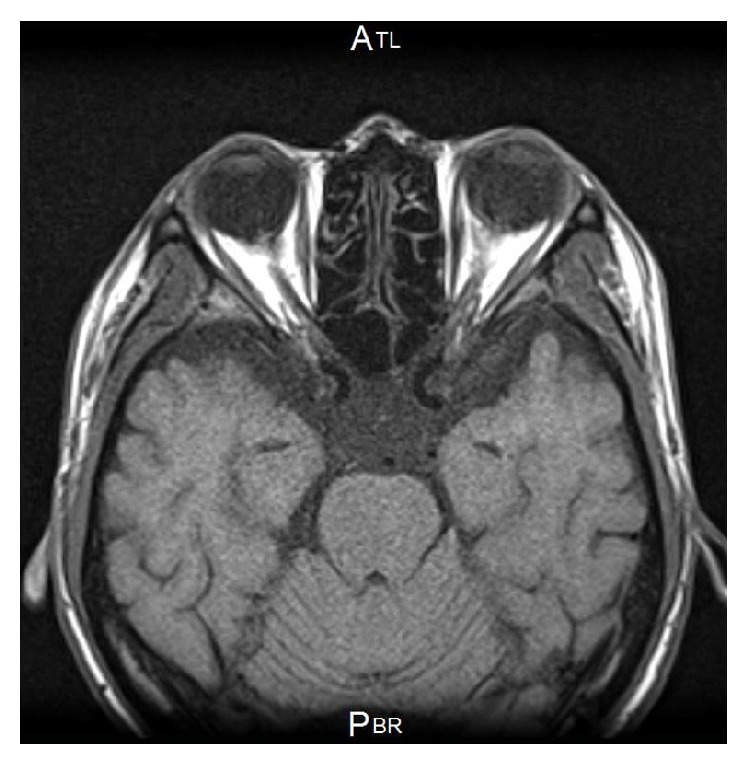
MRI findings in patient with hematopoietic malignancy. The MR images did not detect any abnormalities.

**Figure 4:**
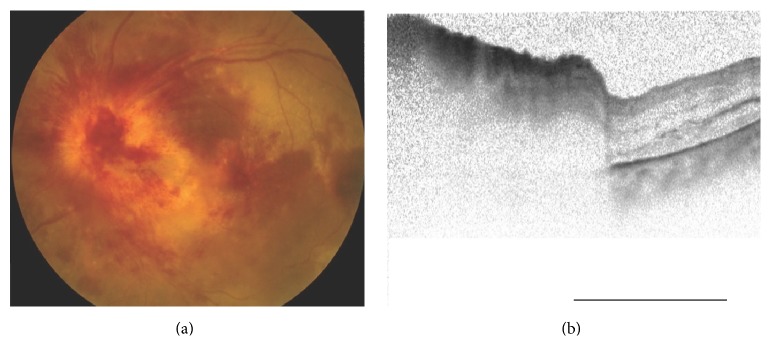
Fundus photograph and OCT image 2 years after the diagnosis of hematopoietic cancer and 2 weeks before death. Optic nerve findings are more severe.

SD-OCT showed a large retinal detachment reaching to the macula and also confirmation of the severe optic disc swelling (Figures 1(c) and 1(d)). The central thickness of the disc was approximately 1263 *μ*m. Fluorescein angiography (FA) showed optic disc hyperfluorescence and serous retinal detachment around the optic disc (Figure 2). Magnetic resonance imaging (MRI) using gadolinium showed that the optic nerves were normal (Figure 3). From these findings, he was diagnosed with ARL with optic nerve involvement. He was informed that radiation therapy of the brain was the only therapy but he refused that. Twenty-four days later, the left visual acuity deteriorated to hand movement at 30 cm, and the ophthalmic examination showed massive retinal and subretinal hemorrhage accompanied by white exudates (Figure 4). The patient's general condition deteriorated and 2 weeks after the last examination, he succumbed to the disease.

### 2.2. Summary of Eight Cases

In our 8 cases, the primary disease was multiple myeloma in 3 cases, acute lymphocytic leukemia (ALL) in 1 case, follicular lymphoma in 1 case, and ARL in 3 cases (Table 1). The orbital invasions were detected during the follow-up examinations, or patients without known cancer had orbital invasion by an undiagnosed cancer. The two cases with optic nerve involvement had undiagnosed ARL, and an internist diagnosed them with ARL from the laboratory data.

**Figure 5:**
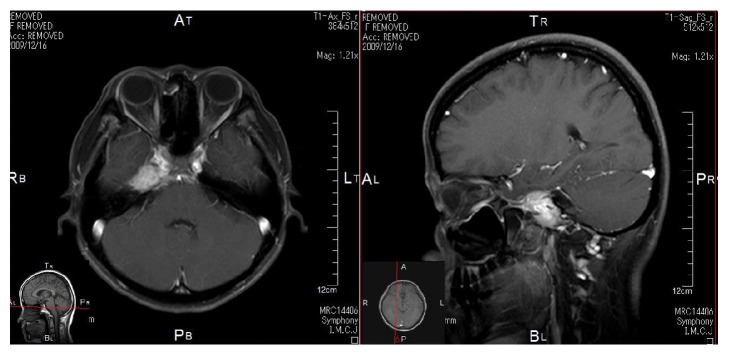
MRI finding of the patient with multiple myeloma who had abducens nerve palsy. MRI images show soft tissue mass enhanced with Ga in the region of the apex of the pyramid.

**Figure 6:**
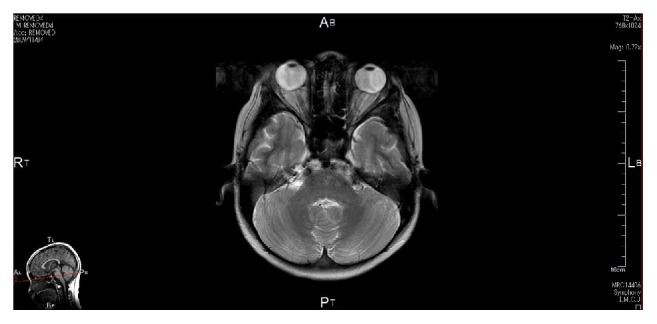
MRI findings of the patient with follicular lymphoma. MRI image shows thickening of the optic nerve sheaths causing “tram track” sign.

The median interval from the diagnosis of cancer and orbital involvement was 4.7 years (1–9 years). Two cases with abducens palsy were detected to have a cranial tumor by MRI (Figure 5). Five cases had no apparent involvement of the optic nerve, and only one case with optic nerve involvement had a thickening of the optic nerve sheath causing a “tram track” sign (Figure 6). Cerebrospinal biopsy revealed tumor cells in two cases and elevated white blood cells in three cases.

Four patients had chemotherapy, and 1 patient improved with steroid therapy and chemotherapy. Three patients had chemotherapy and radiotherapy, and the patient with ALL that had this therapy had a complete remission for 5 years.

Six patients died during the follow-up period of 6 years, and the health of two patients deteriorated rapidly especially the patients with ARL. As has been reported, the prognosis of the patients with hematopoietic cancer is poor [1, 2, 4]. The final decimal visual acuity of the patients with optic nerve infiltration was less than 0.1.

## 3. Discussion

An optic nerve involvement in cases of hematopoietic cancer is very rare. Fung reviewed eight cases of the ophthalmic manifestations in cases of multiple myeloma in his institute over a 15-year period [4]. He reported that eight eyes developed ophthalmic invasions during the 15-year period whereas our results showed that 3 of 8 developed multiple myeloma during a 5-year follow-up period. The neuroophthalmic manifestations were most common in Fung's report. Our results were similar to that of past reports although the improved therapeutic procedures have improved the survival range.

The differential diagnosis for an optic nerve invasion of hematopoietic cancer is neuritis. Because the degree of disc swelling, MRI findings, and laboratory data are different in patients with optic nerve involvement, it is relatively easy to distinguish optic nerve invasion from neuritis (Table 2). The most common sign of an invasion of the optic nerve is a severe disc swelling accompanied by retinal hemorrhage and subretinal fluid and a medical history of cancer. We have evaluated the ophthalmic complications by the medical history, ophthalmoscopic findings of the fundus, FA, OCT findings, MRI, and the results of cerebrospinal pathology. However, not all of the MRI findings and cerebrospinal biopsies showed tumor cells. The results of earlier studies indicated that a positive finding of the first biopsy is 50%, but that of the third biopsy is 90% [17]. However, most individuals refuse multiple biopsies because of the high risk of complications from the biopsy procedures. Nugent recommended diagnostic vitrectomy for manifested optic nerve invasion [7], but a vitrectomy has a risk of compromising the health of the patient because many patients are at the late stage by the time an optic invasion is suspected.

We used OCT to determine whether optic disc alterations were present. Almost all of the OCT images showed massive disc swelling with accompanying subretinal fluid, engorged vessels, and retinal hemorrhage. Although one case had a slightly swollen disc, a disc invasion is generally accompanied by severe disc swelling. The geometric morphometrics of the OCT images clearly confirmed these features. Furthermore, OCT can quantify the degree of severity and identify the structures altered. Compared with past reports, the peripheral retinal nerve fiber layer in our patients was much thicker (800–100 *μ*m) than that in eyes with optic neuritis (about 300 *μ*m) [18]. So we recommended OCT testing for diagnosis because it is noninvasive and can evaluate the degree of disc swelling accurately.

The diagnosis of leptomeningeal metastases usually requires the demonstration of plasma cells in the cerebrospinal fluid or infiltrates in the MR images. It is very difficult to make a diagnosis with only MRI and biopsy because these tests do not always verify tumor cells. Moreover, the orbital leptomeninges can be an anatomical sanctuary where cerebral fluid can be easily stagnated [19]. The positive ratio of tumor cells to normal cells in the optic nerve infiltrates detected by MRI or biopsy was 50% in our cases.

Thus, we need to find other characteristics for a more accurate and early diagnosis. The past report and our results indicate that the optic disc findings and OCT features may be reliable and can be obtained noninvasively. Our findings indicated that severe disc swelling, retinal hemorrhages, and subretinal fluid were strongly associated with optic nerve involvement. Although biopsy and MRI are essential for the diagnosis, patients who have optic nerve involvement generally have progressive disease, and ophthalmologists need to use noninvasive diagnostic tests and avoid diagnostic vitrectomy. Early diagnosis of ophthalmologic involvement can rescue the visual function, and 4 of our cases showed improvements in ocular condition with steroid and systemic therapy. Compared with the cases that had poor ophthalmologic prognosis, the patients with good ophthalmologic prognostic features were associated with longer survival times. Thus, early diagnosis of ophthalmologic involvement is probably important for improving the prognosis of patients with hematopoietic cancer.

## 4. Conclusions

Early detection of ophthalmologic invasion by hematopoietic cancer by ophthalmologists can help preserve the visual function and improve the prognosis of hematopoietic cancer. OCT can help in the diagnosis of ophthalmologic invasion noninvasively.

## Figures and Tables

**Table 1:** Clinical presentation and treatment result in patients with ophthalmologic manifestation of hematopoietic cancer.

Sex	Primary disease	Age of onset	Period of onset	Status	Eye disease	Therapy on eye disease	Visual acuity on first visit	Final visual acuity	Systemic outcome	Ophthalmologic outcome
M	ALL	10 y	3 y	CR	Optic nerve invasion	Chemotherapy	0.6	0.6	Recovered	Recovered
F	MM	39 y	4 y	CR	Sixth-nerve palsy	Chemotherapy	1.2	1.2	Died	Recovered
M	ARL	52 y	1 y	PD	Optic nerve invasion	No treatment	0.05	HM	Died	Deteriorated
F	MM	73 y	9 y	MR	Optic nerve invasion	Steroid pulse therapy	0.05	1.2	Deteriorated	Recovered
M	ARL	43 y	Unknown	No therapy	Optic nerve invasion	Chemo + autologous peripheral stem cell transplantation	1.2	HM	Died	Deteriorated
					Optic nerve invasion		1.2	Unknown		
M	MM	47 y	4.5 y	MR	Sixth-nerve palsy	Chemotherapy	1.2	1.2	Died	Recovered
M	ARL	36 y	Unknown	No therapy	Optic nerve invasion	Chemotherapy	0.05	Unknown	Died	Deteriorated
F	Follicular lymphoma	72 y	4.7 y	PD	Optic nerve invasion	Chemotherapy	0.5	Unknown	Deteriorated	Deteriorated

ALL: acute lymphocytic leukemia; CR: complete response; ARL: AIDS-related lymphoma; PD: progressive disease; MM: multiple myeloma; MR: minor response; HM: hand motion.

**Table 2:** Differential diagnosis of optic nerve invasion.

	Disc swelling	Fundus status	Medical history	MRI findings	Pathology
Optic nerve invasion	Severe	Sometimes accompanied retinal hemorrhage and subretinal fluid	Cancer	Tram truck sign and swelling of optic nerve	Invasion of tumor cell accompanied with infarction
Optic neuritis	In various ways from mild to severe	Mainly disc swelling	Multiple sclerosis	High T2 signal, demyelination, and cloud-like enhancement	Immune response

## References

[1] Yellu M. R., Engel J. M., Ghose A., Onitilo A. A. (2016). Overview of recent trends in diagnosis and management of leptomeningeal multiple myeloma. *Hematological Oncology*.

[2] Oechsle K., Lange-Brock V., Kruell A., Bokemeyer C., De Wit M. (2010). Prognostic factors and treatment options in patients with leptomeningeal metastases of different primary tumors: A Retrospective Analysis. *Journal of Cancer Research and Clinical Oncology*.

[3] Röllig C., Knop S., Bornhäuser M. (2015). Multiple myeloma. *The Lancet*.

[4] Fung S., Selva D., Leibovitch I., Hsuan J., Crompton J. (2005). Ophthalmic manifestations of multiple myeloma. *Ophthalmologica*.

[5] Ko H.-L., Chen C.-L., Chi K.-H. (2009). Frontal skull craniotomy combined with moderate-dose radiotherapy effectively ameliorate a rare case of non-secretory, multiple myeloma with orbital involvement. *World Journal of Surgical Oncology*.

[6] Mansour A. M., Salti H. I. (2001). Multiple myeloma presenting with optic nerve compression. *Eye*.

[7] Nugent A. K., Paulus Y. M., Chan A., Kim J. W., Schwartz E. J., Moshfeghi D. M. (2014). Multiple myeloma recurrence with optic nerve infiltration diagnosed by vitrectomy, immunohistochemistry, and in situ hybridization. *European Journal of Ophthalmology*.

[8] Özdemir S., Tarkan Ö., Tuncer Ü., Surmelioglu Ö., Dogrusoz M., Ergin M. (2013). A case of extramedullary plasmacytoma in the sphenoid sinus with unilateral loss of vision. *Journal of Cranio-Maxillofacial Surgery*.

[9] Pan S. W., Wan Hitam W. H., Mohd Noor R. A., Bhavaraju V. M. K. (2011). Recurrence of multiple myeloma with soft tissue plasmacytoma presenting as unilateral proptosis. *Orbit*.

[10] Riley J. M., Russo J. K., Shipp A., Alsharif M., Jenrette J. M. (2011). Central nervous system myelomatosis with optic neuropathy and intramedullary spinal cord compression responding to radiation therapy. *Japanese Journal of Radiology*.

[11] Shimada Y., Shibuya M., Ohki R., Yoneya S., Nakamura Y. (2006). Bilateral optic neuropathy associated with multiple myeloma. *Journal of Neuro-Ophthalmology*.

[12] Yeung S. N., Paton K. E., Dorovini-Zis K., Chew J. B., White V. A. (2008). Histopathologic features of multiple myeloma involving the optic nerves. *Journal of Neuro-Ophthalmology*.

[13] Yilmaz S. G. U., Ture G., Zengin M. Ö., Talay E., Men S. (2015). Optic nerve and dura mater involvement as the first sign of multiple myeloma. *European journal of ophthalmology*.

[14] Khasraw M., Posner J. B. (2010). Neurological complications of systemic cancer. *The Lancet Neurology*.

[15] Basu S. K., Remick S. C., Monga M., Gibson L. F. (2014). Breaking and entering into the CNS: clues from solid tumor and nonmalignant models with relevance to hematopoietic malignancies. *Clinical and Experimental Metastasis*.

[16] Ravandi F., Cortes J., Estrov Z. (2002). CD56 expression predicts occurrence of CNS disease in acute lymphoblastic leukemia. *Leukemia Research*.

[17] Posner J. B. (1995). *Neurologic Complications of Cancer*.

[18] Hata M., Miyamoto K., Oishi A. (2014). Measurement of retinal nerve fiber layer thickness in eyes with optic disc swelling by using scanning laser polarimetry and optical coherence tomography. *Clinical Ophthalmology*.

[19] Nikaido H., Mishima H., Ono H., Choshi K., Dohy H. (1988). Leukemic involvement of the optic nerve. *American Journal of Ophthalmology*.

